# Evaluating relevance and redundancy to quantify how binary node metadata interplay with the network structure

**DOI:** 10.1038/s41598-019-47717-0

**Published:** 2019-08-06

**Authors:** Matteo Cinelli, Giovanna Ferraro, Antonio Iovanella

**Affiliations:** 1 Department of Enterprise Engineering, University of Rome Tor Vergata, Via del Politecnico, 1, Rome, 00133 Italy; 2 ISC-CNR Uos “Sapienza”, Via dei Taurini, 19, Rome, 00185 Italy

**Keywords:** Applied mathematics, Statistics

## Abstract

Networks are real systems modelled through mathematical objects made up of nodes and links arranged into peculiar and deliberate (or partially deliberate) topologies. Studying these real-world topologies allows for several properties of interest to be revealed. In real networks, nodes are also identified by a certain number of non-structural features or metadata. Given the current possibility of collecting massive quantity of such metadata, it becomes crucial to identify automatically which are the most relevant for the observed structure. We propose a new method that, independently from the network size, is able to not only report the relevance of binary node metadata, but also rank them. Such a method can be applied to networks from any domain, and we apply it in two heterogeneous cases: a temporal network of technology transfer and a protein-protein interaction network. Together with the relevance of node metadata, we investigate the redundancy of these metadata displaying by the results on a Redundancy-Relevance diagram, which is able to highlight the differences among vectors of metadata from both a structural and a non-structural point of view. The obtained results provide insights of a practical nature into the importance of the observed node metadata for the actual network structure.

## Introduction

Networks are used to model interactions across a number of different fields, including social sciences, biology, information technology and engineering. Although the scientific literature predominantly focuses on the topology of the network^1,2^, in several systems nodes themselves possess specific features, which have the potential to shed light on their role in the network^3–7^.

In real networked systems, nodes play at least two different roles: they not only contribute to the construction of the network structure^8,9^, they also carry particular information about themselves^10,11^. Hence, we can identify nodes not only by their connections but also by certain particular features; hereafter referred to as metadata^6,7^. Metadata represent non-structural information that has the potential to display a certain correlation with the observed network structure. Consistently with the increase in the capacity and efficiency of storing data, new networks dataset are also becoming richer in terms of the related amount of metadata. Examples of node metadata include social attributes such as gender^10^, income and group membership^7,11,12^, as well as technical attributes, including product categories for co-purchasing products of online retailers such as Amazon^6,13^. In other terms, once a large set of available node metadata associated to each node of the network has been considered, the following questions can be asked: Which economic indexes should an economist consider as the most relevant in determining new exchanges of goods in a trade network? Which protein functions should a chemical engineer consider as relevant in creating the patterns of a protein-protein interaction network? Which human habits should a social scientist consider as most relevant for the creation of new friendships? Which product features are important for certain items in order for them to be co-purchased?

Building on this further, how can we identify the correlation between the network structure and the node metadata? How can we do this in a computationally efficient way?

As such, detecting the relevance of node metadata becomes key in the investigation of networks, and something that should be tackled to ensure that the information provided is of practical nature and that misleading and time-consuming investigations are avoided. We therefore propose a new method that is able to efficiently compute the relevance of the node metadata by also ranking them. This method is efficient in the sense that, being able to report results independently from the network size, it is not limited by any computational constraints. In this paper, we consider the case in which the node metadata are binary variables, e.g. gender in a social network or protein functions in a protein-protein interaction network, or are variables that are treated in order to be binary, e.g. macroeconomic indexes in a trade network overcoming a given threshold.

The investigation of the relationship between certain binary node metadata and the network topology was performed initially by examining the correlation of the considered binary features across the network edges via the assortativity coefficient^3^. This coefficient, however, doesn’t take into account the microscopic nature of interaction and is preferred in the case of multiple discrete node characteristics or scalar characteristics (like the node degrees). Indeed, in such cases, and conversely from that of binary metadata, the enumeration of each edge type for any node metadata arrangement would be in most of the cases unfeasible. Therefore, in the case of binary node metadata a more detailed approach can be pursued, especially considering that the different link types (called dyads) can be represented in a two-dimensional space. Such approach has been already done, considering undirected networks, in terms of the dyadic effect^4^. The dyadic effect is observed when the number of links between nodes that share a common property is larger than expected by chance^4^. Through the observation of the dyadic effect, two measures, called dyadicity $D$ and heterophilicity $H$, separately denote homogeneous and heterogeneous assortment with respect to a certain binary metadata and measure the degree to which such node metadata correlate with the structure of the network.

In the case of large networks, the methodology proposed by^4^ presents some computational issues based on the notion that, also in the case of binary features, the number of possible configurations increases exponentially with the network size. Therefore, this methodology cannot be practically used for of large networks that possess several node metadata. The scientific literature has tackled this problem in different ways: by simply computing the indexes $D$ and $H$ normalized by random expectations^14,15^; by computing their statistical significance by means of their p-value (therefore computing a null distribution of node metadata)^16,17^; by using entropy-based measures^18,19^, which are numerically hard to compute and whose confidence intervals depends on the number of samples.

In such a framework, our methodology is based on the measures suggested by^4^ but differs from previous contributions in that it focuses on combinatorial arguments deriving from the relationship between the number of featured nodes and the degree sequence of the considered undirected network^20^. Therefore, by exploring the space of configurations generated by binary node metadata, we can discriminate those that are not of interest by comparing the obtained values of $D$ and $H$ with their respective lower bounds, upper bounds and expected values. Moreover, by exploiting the geometric properties of such a space, our method is able to guarantee a high efficiency and scalability, and thus produce results without any computational constraints. We test our methodology on two real networks of heterogeneous nature for which we identify the node metadata that better explain the observed network structure.

To complement the analysis related to the relevance of node metadata, we also consider redundancy in terms of how such metadata are assigned over the network nodes. The interrelation between these two dimensions of relevance and redundancy can be schematised through the introduction of the Redundancy-Relevance diagram (R-R diagram) which provides fruitful insights for the interpretation of networked systems by embedding external sources of information.

## Dyads types and the Dyadic Effect

### Types of dyads

A network can be represented as a graph with *n* nodes and *m* links connecting couples of nodes. We consider a given binary characteristic *c*, which can assume, for simplicity, the values 0 or 1 for each *i* in *n*. The *n* nodes can be divided into two subsets: *n*_1_, the set of nodes with *c*_*i*_ = 1, and *n*_0_, the set of nodes with *c*_*i*_ = 0. Thus, *n* = *n*_1_ + *n*_0_. Consequently three types of dyads, i.e. links and their two end nodes, can be identified in the network: (1 - 1), (1 - 0), and (0 - 0). The amount of each dyad type is labelled as *m*_11_, *m*_10_, *m*_00_, and *m* = *m*_11_ + *m*_10_ + *m*_00_, where *m* is the total number of links in the network. If the characteristics are randomly distributed among the *n* nodes, then any node has an equal chance of having the property 1 and the values of *m*_11_ and *m*_10_ are:

1 $${\bar{m}}_{11}=(\begin{array}{l}{n}_{1}\\ 2\end{array})\delta =\frac{{n}_{1}({n}_{1}-1)}{2}\delta $$

2 $${\bar{m}}_{10}=(\begin{array}{l}{n}_{1}\\ 1\end{array})(\begin{array}{l}{n}_{0}\\ 1\end{array})\delta ={n}_{1}(n-{n}_{1})\delta $$

where $\delta =\frac{2m}{n(n-1)}$ is the network density (i.e. the average probability that two nodes are connected). Additionally, *m*_11_ and *m*_10_ are bounded within specific ranges established, as explained in^20^, by the relationship between the degree sequence *D*_*G*_ of the network and the quantity *n*_1_. **Being** ***d***_***i***_ **the degree of node** ***i*, such bounds can be written as:**

3 $${m}_{11}^{u}=\,{\rm{\min }}(m,\,(\begin{array}{l}{n}_{1}\\ 2\end{array}),\,\lceil \sum _{i\in {D}_{G}^{H}({n}_{1})}\frac{{\rm{\min }}({d}_{i},{n}_{1}-1)}{2}\rceil )$$

4 $${m}_{10}^{u}=\,{\rm{\min }}(m,{n}_{1}{n}_{0},\,{\rm{\min }}(\sum _{i\in {D}_{G}^{H}({n}_{1})}\,{\rm{\min }}({d}_{i},{n}_{0}),\sum _{i\in {D}_{G}^{H}({n}_{0})}\,{\rm{\min }}({d}_{i},{n}_{1})))$$

5 $${m}_{11}^{l}=\,{\rm{\max }}(0,\lfloor \frac{{\sum }_{i\in {D}_{G}^{T}({n}_{1})}{d}_{i}-{\sum }_{i\in {D}_{G}^{H}({n}_{0})}{d}_{i}}{2}\rfloor )$$

6 $${m}_{10}^{l}=(\begin{array}{ll}0 & {\rm{if}}\,{n}_{1}=0,n\\ {\rm{\max }}(1;\sum _{i\in {D}_{G}^{T}({n}_{1})}{d}_{i}-{n}_{1}({n}_{1}-1)) & {\rm{if}}\,{n}_{1}\in (0,n)\end{array})$$

Given a degree sequence *D*_*G*_, by using the quantities *n*_1_ and *n*_0_, which identify the amount of nodes with features 1 and 0 respectively, it is possible to define its head ${D}_{G}^{H}({n}_{1})$ or ${D}_{G}^{H}({n}_{0})$ and its tail ${D}_{G}^{T}({n}_{1})$ or ${D}_{G}^{T}({n}_{0})$ such that ${D}_{G}={D}_{G}^{H}({n}_{1})\cup {D}_{G}^{T}({n}_{0})$ or ${D}_{G}={D}_{G}^{H}({n}_{0})\cup {D}_{G}^{T}({n}_{1})$. In Equation 3, the first term is the number of links in the network, the second term is the number of links in a clique of size *n*_1_, while the third term is the number of links in the sub-graph with *n*_1_ nodes and maximum degree-sum (i.e. with degree sequence ${D}_{G}^{H}({n}_{1})$). In Equation 4, the first term is the number of links in the network, the second term is the number of links in a bipartite graph with partitions of size *n*_1_ and *n*_0_, while the the third term is the minimum between the number of *m*_10_ deriving from the degree partition ${D}_{G}^{H}({n}_{1})\cup {D}_{G}^{T}({n}_{0})$ and the number of *m*_10_ deriving from the degree partition ${D}_{G}^{H}({n}_{0})\cup {D}_{G}^{T}({n}_{1})$. The second term of Equation 5 counts the minimum number of links among the *n*_1_ nodes in the graph deriving from the partition ${D}_{G}^{H}({n}_{0})\cup {D}_{G}^{T}({n}_{1})$, i.e. the amount of *m*_11_ which is realizable from the residual degree of the partition ${D}_{G}^{T}({n}_{1})$. Considering that any connected realization with *n*_1_ ≠ {0, *n*} has at least one *m*_10_, the second term of Equation 6 counts the minimum number of links between the *n*_1_ and *n*_0_ in the case the *n*_1_ are arranged into a clique. The bounds to *m*_00_ can be obtained using the same rationale as that of *m*_11_.

### The dyadic effect

Within the space defined by the bounds, relevant deviations of *m*_11_ and *m*_10_ from the expected values ${\bar{m}}_{11}$ and ${\bar{m}}_{10}$ denote that attribute 1 is not randomly distributed. Such deviations can be computed, in a compact way, through the introduction of two measures called dyadicity *D* and heterophilicity *H*, defined as:

7 $$D=\frac{{m}_{11}}{{\bar{m}}_{11}}$$

8 $$H=\frac{{m}_{10}}{{\bar{m}}_{10}}$$

**Figure 1:**
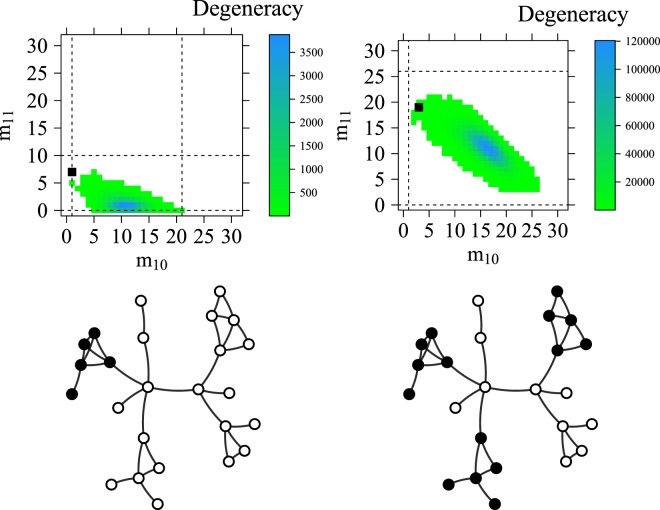
Two instances of the phase diagram with *n*_1_ = 5 and *n*_1_ = 15 embedded within the feasible region defined by the bounds (represented by dashed lines). The two phase diagrams are represented above the two networks from which they are computed. The networks have the same topology with different amounts of *n*_1_ represented as black nodes.

If the distribution of node metadata is dyadic, *D* > 1, it indicates that nodes with the same attributes are more likely to link among themselves than expected in a random configuration. Alternatively, if *D* < 1, the distribution is anti-dyadic, meaning that similar nodes tend to connect less among themselves than expected in a random configuration. The distribution is defined as heterophilic, with a value *H* > 1, highlighting that nodes with the same attributes have more connections to nodes with different features than expected randomly. Otherwise, with a value *H* < 1, the distribution is considered as heterophobic, meaning that nodes with certain attributes have fewer links to nodes with diverse properties than expected randomly. Dyadicity and heterophilicity define a two-dimensional space called *H*–*D* space; a region whereby the way in which binary node metadata are distributed can be investigated. Then, if provided with a set of node metadata, such metadata can be analysed one at the time, computing for each one the deviation of its distribution from random and the correlation with the network structure using the values of *D* and *H*^4^. Moreover, correspondingly with the previous bounds, *D* ranges from ${D}_{min}={m}_{11}^{l}/{\bar{m}}_{11}$ to ${D}_{max}={m}_{11}^{u}/{\bar{m}}_{11}$ and *H* ranges from ${H}_{min}={m}_{10}^{l}/{\bar{m}}_{10}$ to ${H}_{max}={m}_{10}^{u}/{\bar{m}}_{10}$. *D* and *H* consequently allow us to gain some important insights into the meaningfulness of a property shared by a certain number of nodes *n*_1_ ∈ *n*. The correlation between the distribution of a given property *c* and the underlying network topology can be visualized through the phase diagram; an instrument utilized to represent the admissible configurations in a graph. The graph depicted in Fig. 1 is an example of a network with *n* = 25, *m* = 32 and in which *n*_1_ = 5 in one case and *n*_1_ = 15 in the other. The black nodes represent two configurations which are random instances among the $$(\begin{array}{c}n\\ {n}_{1}\end{array})$$ possible ones.

The phase diagram depicts all the admissible combinations of *m*_10_ (x-coordinate) and *m*_11_ (y-coordinate) and each corresponding square collects the number of the assignment of *n*_1_ nodes over the set *n* for every fixed *m*_10_ and *m*_11_. In such diagrams, we can observe how the value of *n*_1_, together with the network topology, is able to affect the shape of the phase diagram, which embeds a wide array of configurations with different degeneracy values. The degeneracy measures the amount of different configurations that provide the same of amount of *m*_11_ and *m*_10_. Investigation into the areas with a high degeneracy is also important since highly degenerative points are close to the expected values of *m*_11_ and *m*_10_, meaning that highly degenerative configurations can be considered as less significant than low degenerative ones. A direct correspondence exists among the *m*_10_ and *m*_11_ axes and, respectively, *H* and *D*. The most typical configurations, i.e. those close to the expected values for which *H* = *D* = 1, are supposed to lie within the core of the phase diagram; consequently, the phase boundaries, being far from the degenerative area, are supposed to indicate atypical configurations.

**Figure 2:**
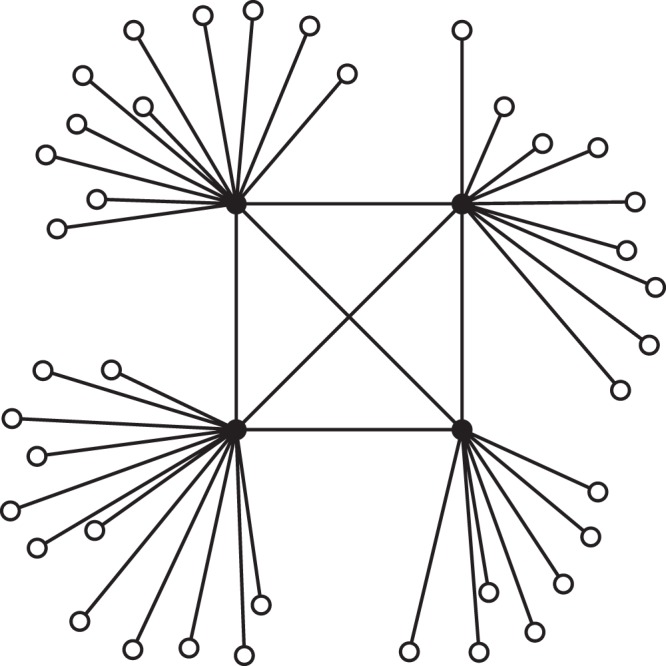
Toy network displaying disassortative mixing but high dyadicity and heterophilicity.

In order to shed light on the differences between the assortativity coefficient *r*^3^ and the metrics *D* and *H* we provide an example to discuss such quantities. We take into account a small network with *n* = 43, *m* = 45 and *n*_1_ = 4, where we have the four higher degree nodes having metadata value *c*_*i*_ = 1, as displayed in Fig. 2. The network displays a strong disassortative mixing with respect to binary metadata (*r* = −0.76), meaning that nodes with same metadata values tend to avoid each other. The analysis of the dyadic effect shows a different and more detailed perspective since the value of dyadicity is *D* = 20 while the value of heterophilicity is *H* = 4.8. By relying only on the value of assortativity, one should expect a higher heterophilicity and a lower dyadicity. In fact, the positive value of *H* confirms the insight from assortativity (i.e. different nodes are interconnected) while the positive value of *D* denotes the presence of tightly interconnected nodes holding *c*_*i*_ = 1, thus adding information to the value of assortativity. In more detail, the disassortative mixing at global level hides the presence of an important local substructure (the so called rich-club^21–23^) in which similar nodes are tightly connected.

The approach of^4^ has been adopted thanks to its peculiarity in bringing together certain endogenous elements related to the topology of the network and some other exogenous elements related to node characterization; however, this only applies to very small networks, e.g. around 50 nodes, due to the difficulty that grows exponentially with the network size. Indeed, this method requires the computation of all the admissible combinations $$(\begin{array}{c}{\boldsymbol{n}}\\ {{\boldsymbol{n}}}_{1}\end{array})$$. The computational complexity of the phase diagram is therefore bounded by such amount of combinations that can be estimated, in the worst case (i.e. when *n*_1_ = *n*/2), to be *O*(2^*n*^) times the number of metadata. Such a value can be computed using the Stirling’s approximation, starting from the binomial coefficient formula (further details are reported in SI).

When real networks with a large number of nodes and several characteristics are considered, this methodology cannot be used. Therefore, a different empirical approach should be taken into account.

## Results

### Quantifying relevance

When we have several node metadata referring to the nodes of a single network, we should take into account two aspects:

i) The comparison of a certain configuration with the related degeneracy area and boundary of the phase diagram may be unfeasible due to computational issues.
ii) For any different value of *n*_1_ the feasible region of the dyadic effect (as well as the shape of the phase diagram) is subject to changes as displayed in Fig. 1.

Therefore, when we aim to evaluate the relevance of a certain set of metadata, we should take into account these two aspects together with the following consideration: the *H*–*D* space is asymmetrical with a unique pivotal point (common for each value of *n*_1_) represented by *H* = *D* = 1 and each of its four internal regions has a different size and meaning, as explained in the previous Section.

**Figure 3:**
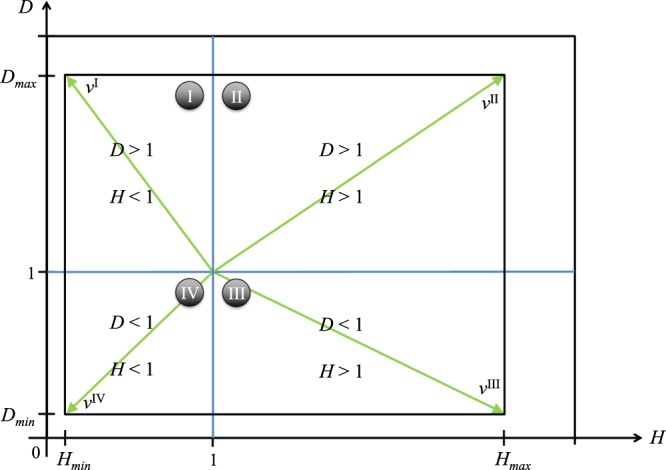
The *H*–*D* space bounded by the values *H*_*min*_, *D*_*min*_, *H*_*max*_, *D*_*max*_ and the four different regions in which a certain configuration of node metadata can lie. The point from which each vector *v*^*i*^ originates is *H* = *D* = 1, i.e. the point in which ${m}_{11}={\bar{m}}_{11}$ and ${m}_{10}={\bar{m}}_{10}$.

Taking into account these three observations, we should evaluate each point in the *H*–*D* space with respect to the boundaries of its own region, and normalize its value with the maximum it can assume in such a region. As shown in Fig. 3, we call region I the Heterophobic-Dyadic region in which the most significant configuration is that with minimum heterophilicity and maximum dyadicity, i.e. the configuration with *H* = *H*_*min*_ and *D* = *D*_*max*_. We call region II the Heterophilic-Dyadic region in which the most significant configuration is that with maximum dyadicity and maximum heterophilicity, i.e. the configuration with *H* = *H*_*max*_ and *D* = *D*_*max*_. We call region III the Heterophilic-Antidyadic region in which the most significant configuration is that with maximum heterophilicity and minimum dyadicity, i.e. the configuration with *H* = *H*_*max*_ and *D* = *D*_*min*_. We call region IV the Heterophobic-Antidyadic region in which the most significant configuration is that with minimum heterophilicity and minimum dyadicity, i.e. the configuration with *H* = *H*_*min*_ and *D* = *D*_*min*_. Once *n*_1_ has been set, the most significant configurations can be represented by vectors, called *v*^*I*^, *v*^*II*^, *v*^*III*^, and *v*^*IV*^, starting from the pivotal point *H* = 1, *D* = 1 and ending in the four vertices of the *H*−*D* space as shown by the green vectors of Fig. 3. These vectors represent the diagonals of the four areas respectively, i.e. the vector of maximum length within the considered region.

**Figure 4:**
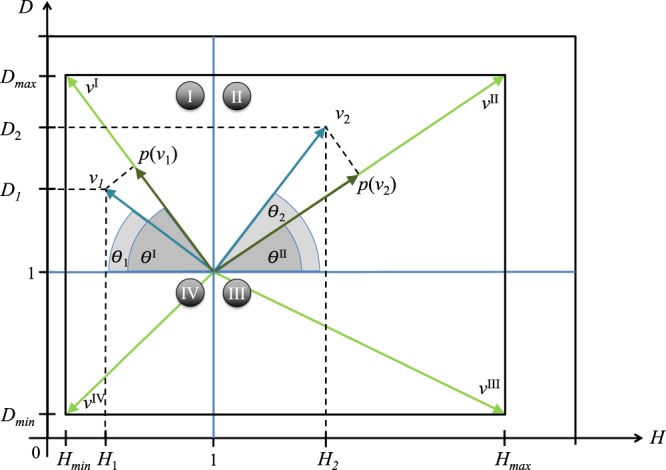
Vectors *v*_1_ and *v*_2_ related to two different binary node metadata with the same amount of *n*_1_. In order to evaluate the relevance of *v*_1_ and *v*_2_ and make a consistent comparison between the two, each of them is projected (*p*(*v*_1_) and *p*(*v*_2_)) onto the diagonal (*v*^I^ and *v*^II^) of the respective space in order to be normalized. The two vectors are supposed to have the same amount of *n*_1_ in order to share the same bounds and thus be compared in the same space.

Any given vector of node metadata can be represented, for a fixed value of *n*_1_, on such a space in the specific region to which it belongs, depending on the values *H* and *D*, and compared with the diagonal related to the considered region. The comparison of each vector with the diagonal of the related region can be performed by projecting the considered vector on such a diagonal and normalizing its value by dividing it by the length of such a diagonal, as shown in Fig. 4. For instance, suppose that we have two characteristics, *c*_1_ and *c*_2_, with an equal amount of *n*_1_ and the corresponding points (*H*_1_, *D*_1_) and (*H*_2_, *D*_2_). In this case it would be clearly difficult to unambiguously identify which one of the characteristics explains better (i.e. is more relevant with respect to) the network structure in absence of the phase diagram.

Therefore we can compute the vectors *v*_1_ and *v*_2_ together with their angles *θ*_1_ and *θ*_2_ with respect to *D* = 1 and project them onto the diagonal of the region in which they lie, obtaining the quantities *p*(*v*_1_) and *p*(*v*_2_). In such a way, we can make a consistent comparison of the vector length with the maximum it can reach in the considered region and compute the significance of the vectors *v*_1_ and *v*_2_ as the ratio ${r}_{1}=\frac{p({v}_{1})}{\parallel {v}^{{\rm{I}}}\parallel }$ and ${r}_{2}=\frac{p({v}_{2})}{\parallel {v}^{{\rm{II}}}\parallel }$. Finally, we can compare *r*_1_ and *r*_2_. The pseudocode related to the proposed methodology is reported in SI (while code can be found at https://github.com/cinhelli). The computational complexity of our method equals the calculation of algebraic relations (that run in constant time) times the number of metadata, thus it can be considered an *O*(1).

In the following section, we apply the proposed methodology to two real-world networks. The first is a temporal network from the technological domain, with node metadata that are treated in order to be binary. The second case is a static network from the biological domain, which has been provided with binary node metadata.

#### Inter-organizational innovation network

Inter-organizational networks have been identified as one of the critical factors in the successful implementation of innovations that allow for the development and achievement of new ideas^24^. Members of inter-organizational networks are linked by joint ventures, licensing arrangements, management contracts, sub-contracting, production sharing and R&D collaboration. We apply the proposed methodology to the case study of an initiative financed by the European Commission called Enterprise Europe Network (EEN), in which nodes are member countries of the network and links represent partnership agreements of technology transfer that exist among them^25^. The members of the network are more than 600 organizations from 54 countries, including universities, research institutes, chambers of commerce, technology centres and development agencies. The parties involved sign a partnership agreement (i.e. a long-term collaboration of technology transfer; hereafter referred to as PA) when the cross-border partner search has been finalized. We analyse the dataset in conjunction with the executing agency of the network (EASME), which covers the span from 2011 to 2014 among the EEN countries. Thus, two nodes, say *i* and *j*, are adjacent through a link if there is at least one connection (a PA formalized by network clients, supported by the EEN members) between them. We analyse the EEN by means of an unweighted graph where the connections between nodes are either present or not. In particular EEN in 2011 has *n* = 48 nodes and *m* = 285 links, EEN in 2012 has *n* = 49 nodes and *m* = 357 links, EEN in 2013 *n* = 51 nodes and *m* = 317 links and, finally, EEN in 2014 has *n* = 52 nodes and *m* = 309 links.

For the analysis of the node characteristics, we refer to the specific node metadata of several indexes from those constituting the Global Innovation Index (GII). The indicators that we take into account are: GDP per capita (GDPpc), Institutions (INST), Human capital and research (HCR), Infrastructure (INFR), Market sophistication (MS), Business sophistication (BS), Knowledge, technology and scientific outputs (KTSO), and Creative outputs (CO). Note that we processed the metadata in order to divide the characteristics into two bins (i.e. we consider dichotomized variables). Considering for each index the average as threshold value, the first bin of size *n*_1_ contains the over-performing EEN countries, i.e. those with an index value greater than the average. The other bin of size *n*_0_ contains the under-performing EEN countries, i.e. those with an index value less than the average. Such a procedure seems appropriate in the case of the EEN, since the considered indicators display a relatively homogenous distribution across the years (see SI). In general, the binarization of metadata is a procedure that is not appropriate for every distribution of scalar quantities. In the case the distribution of metadata is heterogeneous, e.g. it presents a fat-tail, we suggest to adopt other methods for partitioning the distribution such as the characteristic scores and scale (CSS) method described in^26^.

**Figure 5:**
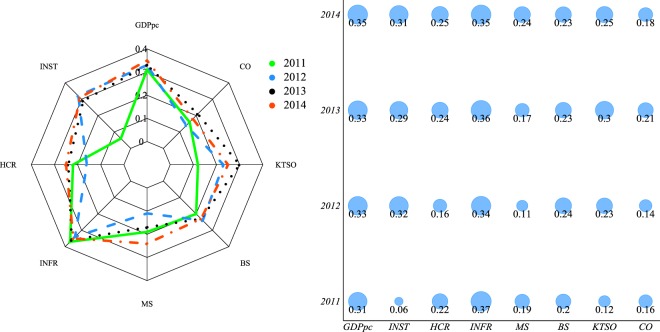
Two different ways of visualizing the relevance of the considered node metadata. The levelling process observed during the four years suggested by the ball plot (left) is then confirmed by the radar plot (right) in which each line corresponds to a year of observation.

The results, by means of the relevance index, are reported in Fig. 5.

The analysis shows that the performance of EEN countries, in terms of innovation and technology transfer, was influenced in the beginning of the observed period by the determinants related to Infrastructure and GDP per capita, meaning that such drivers play a relevant role in the enhancement of the innovation process. Indeed, the innovation capacity at country level depends on the presence of an innovation infrastructure that is strong enough to support research and higher education. In fact, in the late period, the growing importance of investment into human capital and research emerges. This result shows how a knowledge-based strategy is required to encourage innovation through a supportive ecosystem.

From Fig. 5, we observe a levelling process in terms of how relevant are the metadata throughout the four years. This process does not occur for two metadata, namely GDP and INFR, because their influence was predominant since the moment the observation period began. Such evidence suggests that during the process of network formation, GDP and INFR are initially enabling factors while, as time goes by, other indexes start to show their influence. These results confirm that GDP and INFR are facilitating factors for R&D capacities at country level while, over time, a more balanced situation occurs. The levelling process can be attributed to the scope of the EEN initiative, which intends to promote innovation and cooperation within the European Union regardless of any cross-country differences. Further details about EEN and tables of the results are reported in Supplementary Information.

From a more technical point of view, the results of our method display a relatively high accuracy in quantifying the relevance of node metadata. Indeed, we statistically validate the obtained relevance values by computing the probability of finding a higher relevance over a set of 1000 reshuffled vectors of metadata (i.e. vectors with permuted binary entries). Considering the case of EEN in 2011 (Table 1) we note that the higher the relevance score *r* the lower the probability *p*_*r*_ of finding relevant metadata assignments in the set of reshuffled vectors. While computing the probability *p*_*r*_ we also compute two other probabilities *p*_*D*_ and *p*_*H*_ that can give us an idea of the significance of the obtained values. Since the observed configurations are all dyadic and heterophobic *p*_*D*_ is the probability of finding a higher value of *D* while *p*_*H*_ is the probability of finding a lower values of *H*. In the considered case the values of *p*_*D*_ and *p*_*H*_ are in accordance with the relevance score.

**Table 1:** Values associated to the analysis of the dyadic effect for the EEN in 2011.

	*n*_1_	*D*	*H*	*r*	*p*_*D*_	*p*_*H*_	*p*_*r*_
INFR	26.00	1.72	0.82	0.37	0.000	0.000	0.001
GDPpc	26.00	1.60	0.84	0.31	0.010	0.003	0.009
HCR	26.00	1.40	0.87	0.22	0.047	0.008	0.077
BS	27.00	1.36	0.88	0.20	0.047	0.010	0.088
MS	23.00	1.42	0.92	0.19	0.058	0.029	0.164
CO	24.00	1.33	0.91	0.16	0.083	0.017	0.210
KTSO	23.00	1.25	0.93	0.12	0.142	0.039	0.386
INST	26.00	1.11	0.96	0.06	0.289	0.182	0.645

#### Protein-protein interaction network

**Figure 6:**
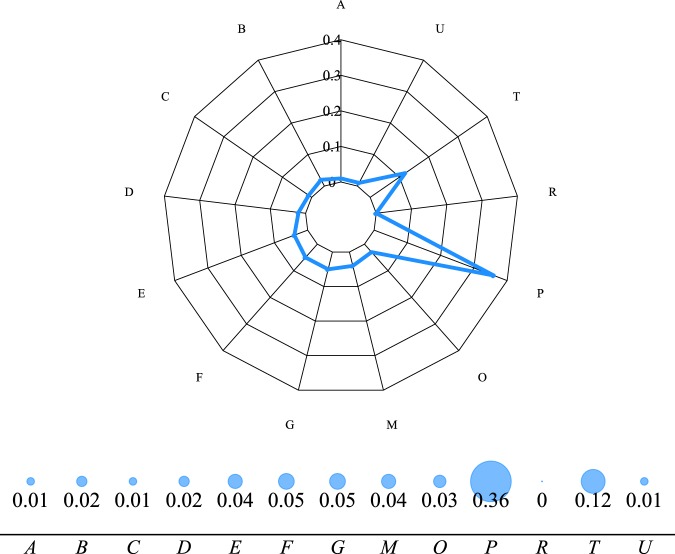
Two different ways of visualizing the relevance of the considered node metadata. In both diagrams it is possible to see how the functional protein category P (protein synthesis) is clearly the more relevant for the observed network structure.

Another real case study is represented by the identification of essential functions of proteins in a protein-protein interaction (PPI) network. A PPI is a mathematical representation of the physical interactions between proteins in a cell. Such a system provides several insights into protein function and allows one to uncover the organizational principles of functional cellular networks. Given that the cells of every organism require the presence of some essential proteins in order to perform their function, the destruction of such proteins entails the death of the organism. Therefore, the recognition of relevant proteins becomes important when the aim is to remove pathogenic organisms for which purpose-specific drugs need to be designed^27^. We take into account the PPI of *Saccharomyces cerevisiae*, which was compiled by^28^ from the data observed by^29^ by identifying 80000 interactions among 5400 proteins and assigning each interaction a confidence level. The considered network dataset corresponds to the largest connected component of this network made up of *m* = 11855 interactions between *n* = 2675 proteins. As node metadata, we consider the protein function as classified by the original Munich Information Center for Protein Sequences (MIPS). The result carried out by our method, i.e. the high relevance of the protein function associated to the category P (protein synthesis) as shown in Fig. 6, is somewhat in accordance with that of^4^ in which such a category displays a high dyadicity (*D* = 16.9, *H* = 1.03). The importance of category P is also confirmed by the correlation between the actual distribution of binary node metadata and the degree of the nodes of such a class (*ρ* = 0.36). In other words, the relevance of the class P is further confirmed by the (merely structural) importance of the associated nodes. Further details about each protein function and tables of the results are reported in Supplementary Information.

Considering the values of *p*_*r*_ reported in Table 2 we note that the higher the relevance score *r* the lower the probability *p*_*r*_ of finding relevant metadata assignments in the set of reshuffled vectors. The values of *p*_*D*_ and *p*_*H*_ may display, in this case, a certain unbalance (e.g. function P) that explains which of the dimensions among *D* and *H* contributes more to the observed relevance.

**Table 2:** Values associated to the analysis of the dyadic effect for the PPI network.

	*n*_1_	*D*	*H*	*r*	*p*_*D*_	*p*_*H*_	*p*_*r*_
P	248	16.90	1.03	0.361	0.000	0.363	0.000
T	240	6.30	1.00	0.115	0.000	0.498	0.218
G	96	9.73	0.60	0.052	0.000	0.001	0.427
F	171	4.66	0.54	0.048	0.000	0.000	0.410
E	95	7.51	0.61	0.039	0.000	0.001	0.429
M	278	2.35	0.58	0.037	0.000	0.001	0.399
O	171	3.30	0.49	0.030	0.000	0.000	0.405
B	98	4.82	0.39	0.023	0.000	0.000	0.444
D	238	1.69	0.43	0.015	0.007	0.000	0.445
C	122	2.68	0.58	0.013	0.001	0.000	0.483
A	51	3.02	0.46	0.008	0.009	0.000	0.560
U	483	1.10	0.63	0.008	0.202	0.000	0.682
R	45	1.46	0.44	0.002	0.156	0.001	0.835

### Relevance and redundancy of node metadata

The process of identifying of relevant binary node metadata has a conceptual interrelation with the procedure of feature selection, used in machine learning to reduce high-dimensional datasets, but it embeds certain structural aspects that derive from the network with which we are provided. The aim of feature selection is to trim data that are either irrelevant or redundant without information losses (we may observe relevant data that are redundant among each other). While the relevance of the considered metadata is computed with the proposed procedure (thus we can discern among relevant vs irrelevant node metadata), the redundancy of such metadata has not been taken into account.

In the case of node metadata, the redundancy can be interpreted as the overlap between the assignments of different metadata values over the nodes of the same network. The concept of redundancy differs from that of degeneracy since the latter is the result of each assignment in terms of edge counts. Indeed, we can’t compute the degeneracy of a certain node metadata assignment, while we can state that a certain *m*_10_ - *m*_11_ couple (i.e. the outcome of the assignment) displays a certain amount of degeneracy.

In our context, and in line with the geometry-based reasoning behind the relevance measure, the redundancy of different node metadata assignments can be defined in terms of cosine similarity among binary vectors of node metadata. Therefore, when two binary vectors of metadata are identical (maximum redundancy), the cosine of the angle related to the dot product of the two vectors will be 1, while when they are completely different (minimum redundancy), the cosine will be 0. Obviously, for a fixed network topology, two completely redundant vectors of binary node metadata will display the same relevance and will result in the same configuration (which, consequently, will display the same degeneracy). However, we may observe different assignments, more or less redundant, generating different *m*_10_ − *m*_11_ couples with different degeneracy and relevance scores.

As our aim is to understand how the metadata relate to the network structure, the redundancy among different metadata carries important information. Indeed, while a couple of metadata with homogeneous nature (for instance, two economic indexes that normally display positive correlation) and high redundancy may not be of interest, a couple of metadata of heterogeneous nature and high redundancy may be of great interest since unrelated features are retained by the same nodes.

**Figure 7:**
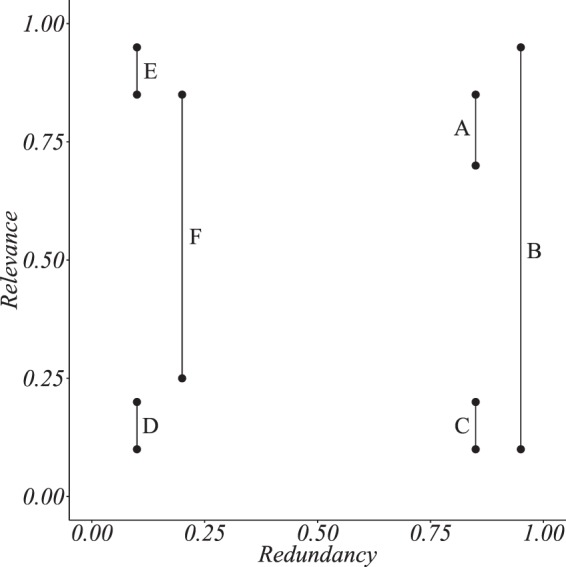
Redundancy-Relevance diagram with six exemplificative configurations. The relevance is computed as explained in the Results Section, while the redundancy is computed via the cosine similarity among different couples of node metadata.

Therefore, the relationship between relevance and redundancy can be schematised in some exemplificative configurations occurring over a Redundancy-Relevance diagram (R-R diagram), as displayed in Fig. 7.

In case A) the couple of node metadata has a high relevance and a high redundancy. The two metadata are both important and they are distributed similarly over the network nodes. Case A) becomes of interest if the two metadata are of heterogeneous nature.

In case B) the two metadata are distributed similarly over the network nodes and one is relevant while the other is not. This may occur because some structurally important nodes, retain the considered metadata and determine the relevance of the related configuration. However, the structural importance of such nodes is intended in a very general sense, since they may have an impact on the measure of relevance for different reasons, such as having high degree or belonging to the same community. Therefore, in case B) it would be of interest to further investigate in which aspects (entries) the two vectors of metadata differ.

Cases C) and D) are not of interest since both the vectors of metadata, either redundant or not, are irrelevant.

In case E) both the metadata are relevant but they are assigned differently over the network nodes. This is an interesting case since nodes with different features (low redundancy) show relevant assignments (high relevance). The considered nodes are different because of the metadata vector and, since the two assignments are relevant, it would be also of interest, in this case, to investigate the structural heterogeneity of such nodes.

In case F) the two vectors of node metadata differ from one another in that one is relevant while the other is not. This last case does not have peculiar implications.

**Figure 8:**
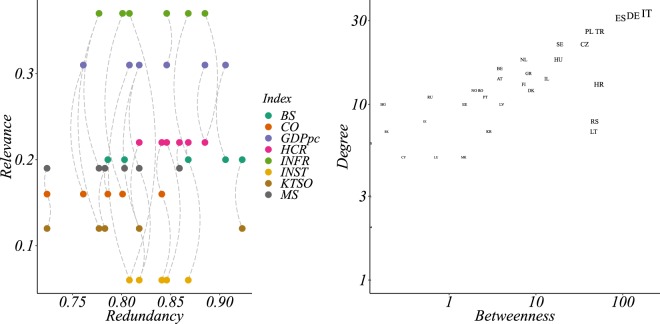
The left panel displays the Redundancy-Relevance diagram for the Enterprise Europe Network during year 2011. The various node metadata are represented with a different color and each couple of indexes is compared in terms of the redundancy of their assignments. A dashed line connects different couples of metadata in order to distinguish the relationship occurring among such couples. The right panel displays the countries of the Enterprise Europe Network during year 2011 over a Degree-Betweenness diagram with logarithmic axes. Such a diagram is able to quantify the importance of different nodes in terms of the size of their neighbourhood (degree) and of information flow (betweenness).

As an example, we compute the R-R Diagram in the case of EEN for the year 2011. The R-R Diagram wouldn’t inherently provide interesting results for the PPI network since, in such a case, there is no overlap among the different binary node metadata (i.e. there are no nodes that belong to multiple categories). The R-R diagram of Fig. 8 (*left*) provides interesting insights into the distribution of metadata over the network nodes. Indeed, we observe how all the considered indexes present a high redundancy in their distribution (high value of cosine similarity) but they can display very different values of relevance. An interesting instance deriving from the R-R Diagram is represented by the relationship between two indexes: INFR and INST. These two indexes are those with the highest and lowest relevance respectively, and they display a high redundancy (0.808) while also displaying the same value of *n*_1_ = 26 and differing in only four entries. Given such high redundancy, the difference in the relevance scores of the two assignments is determined by the properties of only few nodes, which are consequently deemed important from the structural point of view. Such nodes that retain the binary metadata in the case of INFR (the metadata with highest relevance) are Spain, Israel, Italy and Lithuania (ES, IL, IT and LT). This therefore means that we can briefly investigate the structural importance of such nodes over a diagram that embeds two popular centrality measures; namely, degree and betweenness, as shown in Fig. 8 (*right*). On such a diagram these nodes are clearly recognizable, however, their contribution to relevance, in accordance with their structural importance, is not homogeneous. Indeed, Spain and Italy are those contributing the most to the relevance index, being highly central in terms of both degree and betweenness centrality.

## Discussion

In the study of networks, it is important to determine whether certain exogenous features of node, or metadata, impact on the formation of links. This aspect has been studied through the correlation of the network structure with the node metadata, i.e. analysing the presence of assortative mixing. As an alternative to assortative mixing, the correlation of the node metadata with the network structure can be studied at a microscopic level by considering a set of node metadata, their distribution over the network nodes and the resulting amount of dyad types. The departure from random amounts of each dyad type is computed in order to quantify how the network structure and distribution of node metadata are correlated. Thus, such correlation is computed via the measures involved in the study of the dyadic effect, namely heterophilicity (*H*) and dyadicity (*D*). The values of *H* and *D* can be contextualized over a phase diagram, which entails a high computational complexity, or evaluated through other empirical assessments of the *H*–*D* space. The main drawback of such approaches is that they suffer from a certain level of inaccuracy since they fail to consider the extension of the region where the dyadic effect takes place, which changes according to *n*_1_.

Considering the several difficulties in the study of the relationship of the node metadata with the network structure, this paper proposes a new method that is able to provide a ranking of binary node metadata. By applying such an approach, we have been able to detect the metadata that are relevant with respect to the observed network structure. This method is characterized by high efficiency and scalability, which are achieved by exploiting the geometry of the *H*–*D* space in which such metadata are embedded. The efficiency of the method becomes of particular interest when dealing with large networks which are provided with several node metadata or with networks that evolve over time, as we have shown for two real-world networks. The proposed index suffers of certain limitations. The usability of the method is restricted to the case of binary node metadata. However, this constraint can be bypassed through the dichotomization of such metadata with a loss of information that depends on the threshold for dichotomization taken into account.

Additionally, such an index is at global level and lacks of local information. In other words – similarly to other indicators, such as the global clustering coefficient or the assortativity coefficient – it compresses all the information we have about the interaction between the structure and the metadata in a unique index losing other information. Thus, the relevance score should be used to prioritize the analysis of certain metadata against others and should be considered coupled with the respective values of *H* and *D* as well as other measures.

Future work will analyse more in depth the relationship between the network and the node metadata. In particular, a long term challenge could be to consider the interrelations of the structure and metadata in terms of the admissible value of assortativity and, in so doing, prioritise metadata with more accuracy.

Another important contribution presented in this paper is represented by the Redundancy-Relevance diagram. This idea of embedding the redundancy in terms of assignments of node metadata let us evaluate, at the same time, the assignment of node metadata together with their relevance. This result is a new perspective in the evaluation and embedding of external sources of information in complex networks. Thus, while the method that we introduced has a conceptual interrelation with the feature selection process for what concerns the computation of the relevance of data, it differs from feature selection in a fundamental aspect in terms of evaluating redundant data. Such data are normally trimmed in machine learning contexts, where a model needs to be trained, while they are preserved and evaluated, by using the R-R diagram, in our context. The evaluation of the two dimensions of redundancy and relevance without data reduction can be helpful in getting a better understanding and interpretation of the considered system.

## Supplementary information

SI
