# Supplementary material for: Evaluating relevance and redundancy to quantify how binary node metadata interplay with the network structure
Source: Sci Rep. 2019 Aug 6;9:11404. doi: 10.1038/s41598-019-47717-0 (PMC6684645; doi:10.1038/s41598-019-47717-0)
Supplement: Supplementary file 1 — SI [file 41598_2019_47717_MOESM1_ESM.pdf]

Evaluating relevance and redundancy to quantify how binary node metadata interplay with the network structure

Matteo Cinelli[1,2,*,+], **Giovanna Ferraro**[1,+], **and Antonio Iovanella**[1,+]

[1]Department of Enterprise Engineering, University of Rome Tor Vergata, Via del Politecnico, 1 - Rome 00133, Italy
[2]ISC-CNR Uos "Sapienza", Via dei Taurini, 19 - Rome 00185, Italy
[*]matteo.cinelli@uniroma2.it
[+]these authors contributed equally to this work

ABSTRACT

Networks are real systems modelled through mathematical objects made up of nodes and links arranged into peculiar and deliberate (or partially deliberate) topologies. Studying these real-world topologies allows for several properties of interest to be revealed. In real networks, nodes are also identified by a certain number of non-structural features or metadata. Given the current possibility of collecting massive quantity of such metadata, it becomes crucial to identify automatically which are the most relevant for the observed structure. We propose a new method that, independently from the network size, is able to not only report the relevance of binary node metadata, but also rank them. Such a method can be applied to networks from any domain, and we apply it in two heterogeneous cases: a temporal network of technology transfer and a protein-protein interaction network. Together with the relevance of node metadata, we investigate the redundancy of these metadata displaying by the results on a Redundancy-Relevance diagram, which is able to highlight the differences among vectors of metadata from both a structural and a non-structural point of view. The obtained results provide insights of a practical nature into the importance of the observed node metadata for the actual network structure.

Supplementary Information

Computational complexity of the phase diagram

The computation of the phase diagram requires a complete enumeration of all the possible $\binom{n}{n_1}$ combinations of binary metadata over the network nodes. The computational complexity of the phase diagram is bounded by such amount of combinations that can be estimated, in the worst case (i.e. when $n_1 = n/2$), to be $O(2^n)$ times the number of metadata.

Using the Stirling's approximation, $n! \sim \sqrt{2\pi n}(n/e)^n$, we can write:

$$\binom{n}{\frac{n}{2}} = \frac{n!}{(n-\frac{n}{2})!(\frac{n}{2})!} \sim \frac{\sqrt{2\pi n}\,(\frac{n}{e})^n}{(\sqrt{\pi n}\,(\frac{n}{2e})^{\frac{n}{2}})^2} = \frac{\sqrt{2}\,2^n}{\sqrt{\pi n}} \sim O(2^n) \tag{1}$$

In general, the complexity associated to the computation of the phase diagram is strictly related to the ratio between n and n_1 so it is possible to range from linear, yet trivial, cases when $n_1 = 1$ or $n_1 = n$ to exponential cases as that considered for computing the computational complexity.

The necessity to compute and store the combinations associated to the phase diagram causes serious memory issues even for small networks. Considering, for instance, a network with $n = 50$ and $n_1 = 25$ the computation of all the possible combination of binary vectors, that are about $\binom{n}{n_1} \sim 10^{14}$, would require $n \cdot \binom{n}{n_1} = 6 \cdot 10^{15}$ bits that means ~ 0.8 petabytes of memory. This is not the case if we consider $n_1 = 5$ for which there are $\binom{n}{n_1} = 2118760$ configurations to be examined. In summary, the estimation of a limiting amount of nodes is a complicated task that suffers of case dependency and that can become impossible to solve in an exhaustive manner also in the case of networks with few tens of nodes.

PseudoCode

Algorithm 1 Computation of the significance of node metadata

Load a graph G with n nodes and m links
Load a set V of vectors of binary node metadata v_i
for any vector of metadata $v_i \in V$ **do**
  Compute: n_1, H, D, H_{min}, D_{min}, H_{max}, D_{max}
  Compute:

- $v^{\mathrm{I}} = (1-H_{min}, D_{max}-1)$, $\|v^{\mathrm{I}}\| = \sqrt{(1-H_{min})^2+(D_{max}-1)^2}$ and $\theta^{\mathrm{I}} = arccos\left(\frac{1-H_{min}}{\|v^{\mathrm{I}}\|}\right)$
- $v^{\mathrm{II}} = (H_{max}-1, D_{max}-1)$, $\|v^{\mathrm{II}}\| = \sqrt{(H_{max}-1)^2+(D_{max}-1)^2}$ and $\theta^{\mathrm{II}} = arccos\left(\frac{H_{max}-1}{\|v^{\mathrm{II}}\|}\right)$
- $v^{\mathrm{III}} = (H_{max}-1, 1-D_{min})$, $\|v^{\mathrm{III}}\| = \sqrt{(H_{max}-1)^2+(1-D_{min})^2}$ and $\theta^{\mathrm{III}} = arccos\left(\frac{H_{max}-1}{\|v^{\mathrm{III}}\|}\right)$
- $v^{\mathrm{IV}} = (1-H_{min}, 1-D_{min})$, $\|v^{\mathrm{IV}}\| = \sqrt{(1-H_{min})^2+(1-D_{min})^2}$ and $\theta^{\mathrm{IV}} = arccos\left(\frac{1-H_{min}}{\|v^{\mathrm{IV}}\|}\right)$

  if $H \equiv 1$ and $D \equiv 1$ **then**
    $r_i = 0$ ▷ i.e. the vector v_i has no significance
  end if
  if $(H,D) \in I$ **then** ▷ i.e. $H < 1$ and $D > 1$
    $v_i = (1-H, D-1)$, $\|v_i\| = \sqrt{(1-H)^2+(D-1)^2}$ and $\theta_i = arccos\left(\frac{1-H}{\|v_i\|}\right)$
    $p(v_i) = \langle v_i, v^I \rangle$
    $r_i = \frac{p(v_i)}{\|v^I\|}$
  end if
  if $(H,D) \in II$ **then** ▷ i.e. $H > 1$ and $D > 1$
    $v_i = (H-1, D-1)$, $\|v_i\| = \sqrt{(H-1)^2+(D-1)^2}$ and $\theta_i = arccos(\frac{H-1}{\|v_i\|})$
    $p(v_i) = \langle v_i, v^{II} \rangle$
    $r_i = \frac{p(v_i)}{\|v^{II}\|}$
  end if
  if $(H,D) \in III$ **then** ▷ i.e. $H > 1$ and $D < 1$
    $v_i = (H-1, 1-D)$, $\|v_i\| = \sqrt{(H-1)^2+(1-D)^2}$ and $\theta_i = arccos(\frac{H-1}{\|v_i\|})$
    $p(v_i) = \langle v_i, v^{III} \rangle$
    $r_i = \frac{p(v_i)}{\|v^{III}\|}$
  end if
  if $(H,D) \in IV$ **then** ▷ i.e. $H < 1$ and $D < 1$
    $v_i = (1-H, 1-D)$, $\|v_i\| = \sqrt{(1-H)^2+(1-D)^2}$ and $\theta_i = arccos(\frac{1-H}{\|v_i\|})$
    $p(v_i) = \langle v_i, v^{IV} \rangle$
    $r_i = \frac{p(v_i)}{\|v^{IV}\|}$
  end if
end for
Sort r in non decreasing order

Enterprise Europe Network

Explanation of the indexes

For the node metadata, we refer to several indexes from those constituting the Global Innovation Index (GII) reports. The GII reports are generally considered a leading reference on innovation and they are co-published by Cornell University, INSEAD and the World Intellectual Property Organization (WIPO). The reports are published annually and available at the web address http://www.globalinnovationindex.org. The indicators that we take into account are: GDP per capita (GDP), Institutions (INST), Human capital and research (HCR), Infrastructure (INFR), Market sophistication (MS), Business sophistication (BS), Knowledge, technology and scientific outputs (KTSO), and Creative outputs (CO).

In particular, for each country, we refer to the GDP per capita in PPP (purchasing power parity) in dollars, as extracted from the World Bank World Development Indicators databases. We also consider the value score for seven different indexes, defined as pillars in the GII reports. Indeed, the GII refers to two sub-indices: the Innovation Input Sub-Index and the Innovation Outputs Sub-Index, each built around these pillars. The Innovation Input Sub-Index has five enabler pillars: Institutions (INST), Human capital and research (HCR), Infrastructure (INFR), Market sophistication (MS), and Business sophistication (BS). Enabler pillars are related to aspects of the environment that are favourable to innovation within an economy. The other two pillars concern the innovation activities within an economy and are related to innovation outputs. They are: Knowledge, technology and scientific outputs (KTSO) and Creative outputs (CO). All the formal descriptions on GII, as well as its constituting indexes, are reported in the official reports available at the web address: http://globalinnovationindex.org. **In order to compute the relevance of node metadata we binarize the values of the considered indicators, considering countries over-performing ($c_i = 1$) and under-performing ($c_i = 0$) with respect to the mean of a certain indicator. Such a procedure seems appropriate in the case of the EEN, since the considered indicators display a relatively homogenous distribution across the years, as shown in Figure 1. In general, the binarization of metadata is a procedure that is not appropriate for every distribution of scalar quantities. In the case the distribution of metadata is heterogeneous, e.g. it presents a fat-tail, we suggest to adopt other methods for partitioning the distribution such as the characteristic scores and scale (CSS) method.**

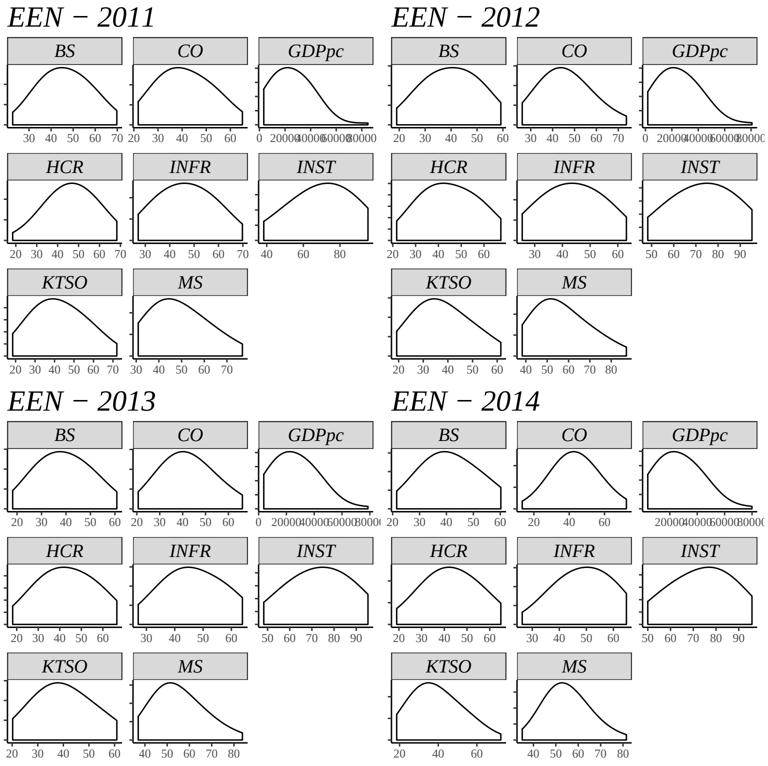

Figure 1. Distribution of the indicators constituting the GII index across the countries in EEN for the years 2011, 2012, 2013 and 2014.

Tables

	n_1	D	H	D_{max}	H_{max}	D_{min}	H_{min}	r
INFR	26.00	1.72	0.82	2.72	1.97	0.00	0.12	0.37
GDPpc	26.00	1.60	0.84	2.72	1.97	0.00	0.12	0.31
HCR	26.00	1.40	0.87	2.72	1.97	0.00	0.12	0.22
BS	27.00	1.36	0.88	2.62	1.99	0.00	0.10	0.20
MS	23.00	1.42	0.92	3.08	1.96	0.00	0.14	0.19
CO	24.00	1.33	0.91	2.95	1.96	0.00	0.17	0.16
KTSO	23.00	1.25	0.93	3.08	1.96	0.00	0.14	0.12
INST	26.00	1.11	0.96	2.72	1.97	0.00	0.12	0.06

Table 1. EEN 2011 mean

	n_1	D	H	D_{max}	H_{max}	D_{min}	H_{min}	r
INFR	25.00	1.65	0.90	2.69	1.96	0.00	0.22	0.34
GDPpc	25.00	1.65	0.92	2.69	1.96	0.00	0.22	0.33
INST	28.00	1.51	0.83	2.41	2.00	0.00	0.14	0.32
BS	27.00	1.40	0.87	2.51	1.98	0.00	0.17	0.24
KTSO	21.00	1.57	1.00	3.09	2.00	0.00	0.14	0.23
HCR	26.00	1.29	0.93	2.59	1.97	0.00	0.19	0.16
CO	25.00	1.25	0.94	2.69	1.96	0.00	0.22	0.14
MS	24.00	1.22	0.94	2.79	1.96	0.00	0.22	0.11

Table 2. EEN 2012 mean

	n_1	D	H	D_{max}	H_{max}	D_{min}	H_{min}	r
INFR	25.00	1.81	0.90	3.11	1.96	0.00	0.13	0.36
GDPpc	25.00	1.73	0.93	3.11	1.96	0.00	0.13	0.33
KTSO	21.00	1.74	1.02	3.66	2.02	0.00	0.06	0.30
INST	28.00	1.61	0.87	2.73	1.98	0.00	0.09	0.29
HCR	26.00	1.44	0.93	2.98	1.96	0.00	0.13	0.24
BS	28.00	1.42	0.86	2.73	1.98	0.00	0.09	0.23
CO	25.00	1.38	0.94	3.11	1.96	0.00	0.13	0.21
MS	24.00	1.27	1.01	3.24	1.97	0.00	0.11	0.17

Table 3. EEN 2013 mean

Protein-Protein Interaction Network

MIPS Functional Categories

MIPS Functional Categories of *Saccharomyces cerevisiae*.

	n_1	D	H	D_{max}	H_{max}	D_{min}	H_{min}	r
INFR	25.00	1.65	0.90	2.69	1.96	0.00	0.22	0.34
BS	27.00	1.40	0.87	2.51	1.98	0.00	0.17	0.24
GDPpc	25.00	1.65	0.92	2.69	1.96	0.00	0.22	0.33
INST	28.00	1.51	0.83	2.41	2.00	0.00	0.14	0.32
CO	25.00	1.25	0.94	2.69	1.96	0.00	0.22	0.14
KTSO	21.00	1.57	1.00	3.09	2.00	0.00	0.14	0.23
HCR	26.00	1.29	0.93	2.59	1.97	0.00	0.19	0.16
MS	24.00	1.22	0.94	2.79	1.96	0.00	0.22	0.11

Table 4. EEN 2014 mean

Category	Description	Original MIPS category
E	energy production	energy
G	aminoacid metabolism	aminoacid metabolism
M	other metabolism	all remaining metabolism categories
P	translation	protein synthesis
T	transcription	transcription, but without subcategory "transcriptional control"
B	transcriptional control	subcategory "transcriptional control"
F	protein fate	protein fate (folding, modification, destination)
O	cellular organization	cellular transport and transport mechanisms
A	transport and sensing	categories "transport facilitation" and "regulation of / interaction with cellular environment"
R	stress and defense	cell rescue, defense and virulence
D	genome maintenance	DNA processing and cell cycle
C	cellular fate / organization	categories "cell fate" and "cellular communication / signal transduction" and "control of cellular organization"
U	uncharacterized	categories "not yet clear-cut" and uncharacterized

Table 5. MIPS metadata

Tables

	n_1	D	H	D_{max}	H_{max}	D_{min}	H_{min}	r
P	248	16.90	1.03	44.67	5.19	0.00	0.00	0.36
T	240	6.30	1.00	46.94	5.25	0.00	0.00	0.12
F	171	4.66	0.54	77.51	5.98	0.00	0.00	0.05
G	96	9.73	0.60	167.16	7.30	0.00	0.00	0.05
E	95	7.51	0.61	168.84	7.32	0.00	0.00	0.04
M	278	2.35	0.58	37.56	4.84	0.00	0.00	0.04
O	171	3.30	0.49	77.51	5.98	0.00	0.00	0.03
B	98	4.82	0.39	163.83	7.24	0.00	0.00	0.02
D	238	1.69	0.43	47.54	5.27	0.00	0.00	0.02
A	51	3.02	0.46	241.02	9.08	0.00	0.00	0.01
C	122	2.68	0.58	125.16	6.72	0.00	0.00	0.01
U	483	1.10	0.63	15.98	3.08	0.00	0.00	0.01
R	45	1.46	0.44	240.88	9.51	0.00	0.00	0.00

Table 6. Protein-Protein Interaction Network
